# Closed-loop carbon management strategies for climate and energy-resilient India

**DOI:** 10.1016/j.isci.2025.113509

**Published:** 2025-09-05

**Authors:** Piyali Majumder, Arnab Dutta

**Affiliations:** 1 Chemistry Department, Indian Institute of Technology Bombay, Powai, Mumbai 400076, India; 2 Centre for Climate Studies, Indian Institute of Technology Bombay, Powai, Mumbai 400076, India; 3 National Centre of Excellence for Carbon Capture, Utilization, and Storage, Indian Institute of Technology Bombay, Powai, Mumbai 400076, India

**Keywords:** Applied sciences, Energy Resources, Energy management

## Abstract

Climate change, primarily driven by excessive CO_2_ emissions, poses urgent global challenges. India contributes over 2,600 Mt CO_2_ annually, with ∼80% originating from hard-to-abate sectors, such as power, cement, petrochemical, chemical, and steel. This review highlights the importance of carbon capture, utilization, and storage (CCUS) and carbon dioxide removal (CDR) technologies as critical pathways to align India’s growth with climate goals. India prioritizes point-source capture from flue gases, reflecting its concentrated emission profile. Effective carbon management models must generate high-value marketable products, including fuels, fertilizers, aggregates, and construction materials, supporting a closed-loop carbon cycle. Mineralization pathways enable CO_2_-based building materials to strengthen Smart City and infrastructure initiatives, while CO_2_-derived fertilizers enhance agricultural productivity and food security. The manuscript also examines India’s emerging carbon market, recommending balanced compulsory and voluntary mechanisms, carbon credit incentives, and utilization-driven byproducts. Collectively, it provides an integrated CCUS framework for India that couples emission reduction with economic viability and offers scalable insights for other developing economies.

## Graphical abstract

**Figure:**
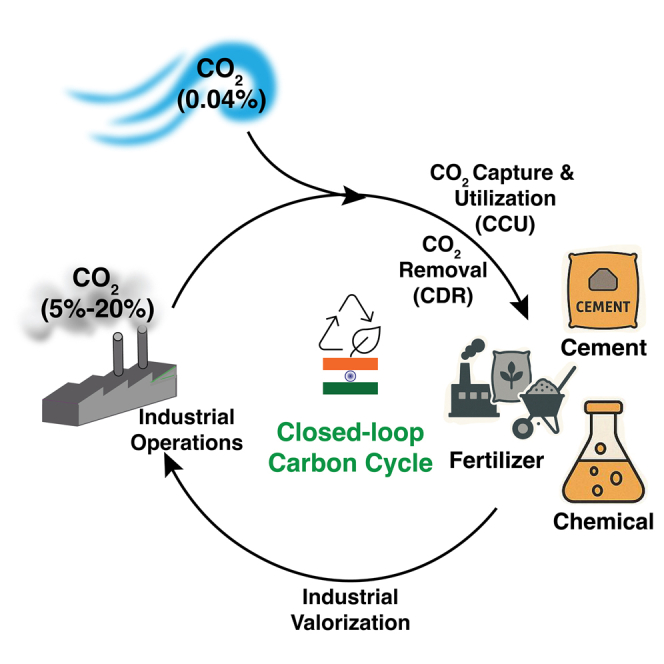

## Introduction

**Figure 1:**
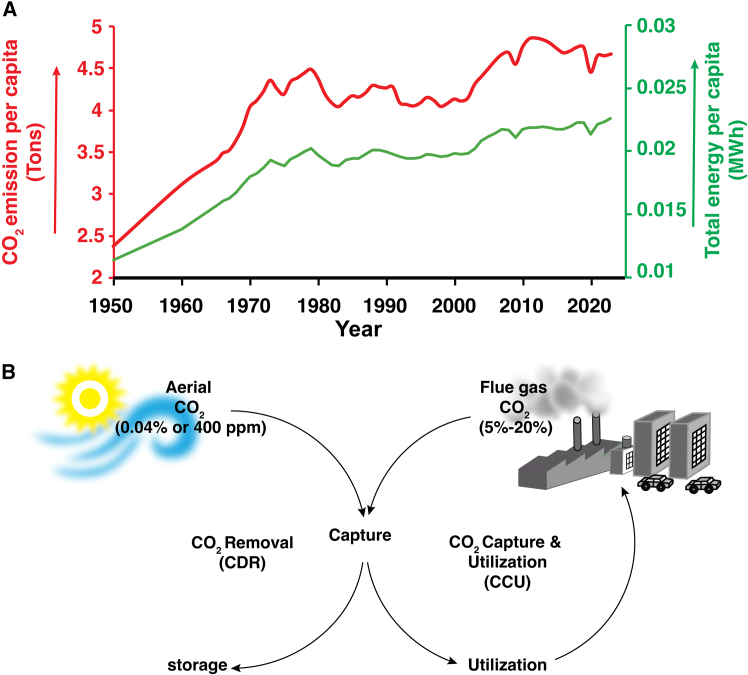
The energy usage-linked anthropogenic CO_2_ emission and applications of CCU CDR for CO_2_ removal (A) The comparative trend of global energy usage and anthropogenic CO_2_ emission per capita since 1950.^6^ (B) The CO_2_ capture (from both atmospheric 0.04% and industrial 5%–20% v/v CO_2_), utilization (CCU) and CO_2_ removal (CDR) strategies and their influence on the carbon cycle.

The considerable augmentation in worldwide energy consumption, attributable to the rising demographic statistics and significant advancements in quality of life across diverse nations, has culminated in a remarkable escalation in the necessity for energy resources, with a predominant portion of this necessity being fulfilled through the utilization of fossil fuels, which are acknowledged for their carbon-intensive properties.^1^ The combustion processes entailed in the application of these carbon-based energy resources give rise to the emission of considerable quantities of greenhouse gases (GHGs) into the Earth’s atmosphere, thereby exacerbating the current climate change crisis that poses grave dangers to the natural ecological equilibrium (Figure 1A).^2^ The substantial volume of carbon dioxide (CO_2_) and methane (CH_4_) emissions, coupled with their inherent stability, has led to their classification as the foremost greenhouse gases (GHGs), notwithstanding the existence of other atmospheric constituents, including water vapor and ozone.^3^^,^^4^ While it is significant to note that CH_4_ demonstrates a markedly elevated immediate greenhouse effect, roughly 80 times that of CO_2_ when assessed over a two-decade period, it is also distinguished by a relatively brief atmospheric longevity and enhanced industrial applicability concerning its utilization.^5^ In contrast, CO_2_ exhibits a propensity to persist in the atmosphere for protracted durations, thereby necessitating the urgent development and execution of efficacious removal strategies designed to mitigate its adverse environmental impacts.

The urgency surrounding carbon sequestration arises from the accelerating pace of anthropogenic carbon dioxide (CO_2_) emissions since the 1960s. The advent of the industrialization era, coupled with overgrowing population demand, deforestation, and overreliance on fossil fuels, is considered the prime reason behind this phenomenon.^7^ The accumulation of CO_2_ in the atmosphere adversely influenced the global average temperatures (contributing 53% of the global greenhouse effect),^8^ triggering intensified heatwaves, polar ice melting, ocean acidification, and biodiversity collapse. Such a calamitous climate change is now broadly recognized as one of the crucial global challenges in front of human civilization, which demands not only the mere reduction of our future carbon emissions, but also a serious effort to remove the already existing CO_2_ pool in the atmosphere.

Restricting the rise of global average temperature to 1.5°C is widely considered a threshold to avoid irreversible climate tipping points, as reported by the Intergovernmental Panel on Climate Change (IPCC).^9^ It will require both rapid decarbonization and large-scale deployment of carbon removal strategies to practically implement such climate control effects. These strategies, including natural sinks and engineered carbon capture systems, form a crucial bridge between short-term mitigation and long-term climate stabilization efforts. Without such interventions, the planet risks cascading climate feedback loops that could significantly undermine social, economic, and ecological systems worldwide.^10^^,^^11^

One of the principal strategies for decarbonization, known as carbon dioxide removal (CDR), is characterized as the deliberate procedure that involves the direct extraction of atmospheric carbon dioxide (CO_2_), which is thereafter subjected to irreversible sequestration within geological formations, thus facilitating a significant diminution in the total concentration of CO_2_ present in the atmosphere.^12^ In a concurrent methodology, carbon capture and utilization (CCU) is predicated upon the deliberate extraction of emitted CO_2_ from point sources (typically varying between 5 and 20% (v/v) CO_2_ across the leading hard-to-abate sectors) prior to its release into the atmosphere (Figure 1B). Thereafter, this captured CO_2_ is converted into commercially feasible products, thus promoting the realization of a carbon-neutral or net-zero emissions paradigm. At present, the atmospheric concentration of carbon dioxide (CO_2_) has surpassed the threshold of 430 parts per million (ppm) (>0.04%), demonstrating a consistent annual increase of approximately 2.0 ppm.^13^ A significant threat to the equilibrium of our climate is presented by such a rate of increase in atmospheric CO_2_, which necessitates prompt action to address the situation. To achieve stabilization of the current climatic conditions, it is imperative to enact strategies that will lower this concentration to 350 ppm or below, which requires the extraction of historical CO_2_ emissions estimated at around 780 million tons, an amount that equates to nearly 100 ppm, thereby underscoring the essential need for the advancement of carbon-negative CDR technologies to fulfill these goals.

**Figure 2:**
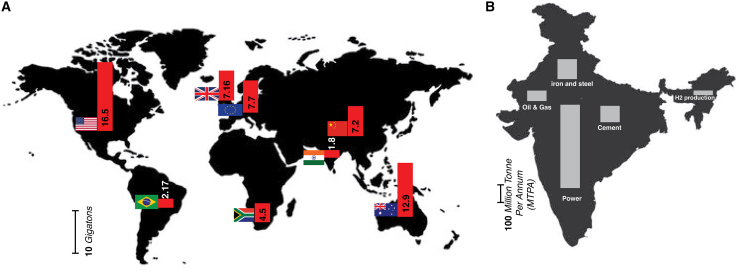
The country-wise global CO_2_ emission and sector-wise CO_2_ emission in India (A) Global scenario on country-wise CO_2_ emissions in 2022.^24^ (B) Sector-wise CO_2_ emission in India in 2020.^23^

India is positioned as the third-largest consumer of energy on an international level, with an estimated 75% of its energy demands fulfilled by fossil fuel sources.^14^^,^^15^ This reliance designates the nation as one of the largest emitters of CO_2_ on a global scale (Figure 2A).^16^^,^^17^^,^^18^ The industrial sectors, most notably power generation, steel manufacturing, petrochemical production, cement fabrication, and chemical processing, constitute the essential framework of India’s economic advancement.^19^ Nevertheless, these sectors are often classified as “hard-to-abate” due to the significant technological and economic challenges that are intrinsic to the decarbonization of their operations. According to the results articulated in the carbon capture, utilization, and storage (CCUS) report disseminated by NITI Aayog, the power and industrial sectors within India collectively emitted approximately 1,600 million tons per annum (mtpa) of CO_2_ in the year 2020, which represented nearly 60% of the country’s overall emissions, totaling 2,600 mtpa. The remaining 40% of emissions are ascribed to distributed sources, including agriculture, transportation, and building operations.^20^ Within the industrial domains, power generation stands out as the principal contributor to CO_2_ emissions, predominantly influenced by electricity generation from coal.^21^ The steel industry similarly plays a crucial role in contributing to greenhouse gas emissions and is projected to experience growth while persistently relying on fossil fuel sources. The production of cement constitutes another notable contributor to emissions, stemming from both energy utilization and activities associated with the manufacturing process.^22^ Similarly, the chemical and petrochemical sectors are responsible for substantial emissions due to their elevated energy intensity. Collectively, these sectors are accountable for over 70%–80% of India’s total CO_2_ emissions, underscoring a crucial area for climate mitigation initiatives (Figure 2B).^23^ In the year 2019 alone, these industrial sectors contributed around 600 million tonnes to national CO_2_ emissions.^23^ Within these sectors, the steel industry accounted for around 242 Mt, followed by the cement industry at 170 Mt, and the chemical sector at 137 Mt.^23^ These three industries alone represent a significant share of India’s total industrial emissions, making them high-impact targets for decarbonization. In this framework, the deployment of CCUS technologies within these critical sectors could serve as a transformative strategy. By enabling the extraction and ensuing repurposing or permanent storage of CO_2_ at the point of origin, CCUS presents a viable pathway for India to markedly reduce its industrial emissions and advance toward its commitments to environmental sustainability and climate change mitigation.

India is actively piloting several CDR and CCU technologies within industrial premises to align with its economic trajectory and net-zero ambitions across the sectors. Notable examples include Tata Steel’s CCU pilot in Jamshedpur (captures CO_2_ from flue gas at 5.0 TPD scale),^25^ NTPC’s flue gas CO_2_ capture trials at thermal power plants at Vindhyachal (captures 20.0 TPD CO_2_ and converts it to equivalent amount of methanol),^26^ and the operational CCU facility at the Tuticorin Alkali Chemicals plant^27^ (converts captured CO_2_ into soda ash). Additionally, Jindal Steel has initiated on-site CO_2_ capture and reuse efforts near Angul, Odisha.^28^ Geological studies have identified India’s Deccan Traps as a significant basalt formation suitable for long-term CO_2_ mineralization, offering vast domestic sequestration capacity.^29^ Compared to the U.S. and EU, where large-scale carbon capture is driven by mature infrastructure and subsidies, India’s approach focuses on cost-effective, scalable deployment to match its rapid growth and industrial expansion. These efforts, if accelerated through policy incentives and private investment, can ensure an effective decarbonization without hindering India’s economic development.

India has adopted an assertive and proactive approach in the international endeavor to combat climate change, thereby reinforcing its dedication to a sustainable future through significant commitments articulated under the Paris Agreement and reaffirmed at COP26 in Glasgow. Among these commitments, the nation has pledged to diminish the carbon intensity of its economy by 45% by the year 2030 and to attain net-zero emissions by 2070—aspirational objectives that exemplify both the magnitude of the challenge and India’s resolve to assume a leadership role with intent.^30^ Achieving these objectives necessitates more than mere intent; it requires a methodical and cohesive framework for carbon management. This encompasses the implementation of progressive policies and the enhancement of the utilization of CDR and CCU technologies. Such instruments are pivotal in facilitating India’s sustained economic advancement while concurrently achieving substantial reductions in emissions—an equilibrium that lies at the core of the nation’s climate policy. An integral component of this transition entails the deployment of hybrid carbon capture technologies that possess the capability to operate across a wide array of CO_2_ concentrations, spanning from the minimal levels present in ambient air (approximately 0.04%) to the considerably elevated concentrations characteristic of industrial flue gases (oscillating between 20% and 60%). These technologies are not only scientifically intricate but also indispensable for mitigating emissions from both dispersed and concentrated sources. Encouragingly, there is a thriving culture of innovation in this domain. Indian start-ups are at the forefront, developing robust and economically viable carbon capture solutions that are specifically designed to address the distinct requirements of the country’s industrial framework.^31^ These advancements transcend mere technical accomplishments; they serve as formidable emblems of India’s determination to confront climate change via indigenous, sustainable innovation. As India advances in its climate trajectory, these initiatives will be instrumental in transforming national pledges into quantifiable progress for the environment.

Addressing the dual imperative of satisfying escalating energy requirements while simultaneously alleviating the urgent challenges posed by climate change necessitates a holistic and multifarious approach. In India, the strategic deployment of CDR and CCU technologies is deemed essential, contingent upon the reinforcement of robust policy frameworks and persistent innovation. By emphasizing the decarbonization of sectors that present significant difficulties in abatement and directing investments toward scalable CO_2_ removal technologies that are fundamentally supported by renewable energy, India can attain notable progress in its climate-related objectives. Significantly, this trajectory allows the nation to synchronize with its developmental ambitions, thereby illustrating that economic development and environmental responsibility can coexist in a mutually reinforcing manner.

## CO_2_ utilization technologies

The necessity to alleviate global carbon emissions in order to achieve the 1.5°C threshold delineated by the Paris Agreement highlights the pressing demand for extensive CO_2_ extraction methodologies. Modern evaluations assert that around 7 to 9 billion metric tons of carbon dioxide must be abated from the atmosphere on an annual basis by the year 2050.^32^ This equates to a daily removal requirement of around 3 million tons, translating to an astounding volume of 500,000 billion liters of pure CO_2_.^33^ However, achieving this scale of removal becomes even more challenging when targeting atmospheric CO_2_. Due to its low concentration of approximately 0.04%, atmospheric CO_2_ removal necessitates the processing of air volumes 2,500 times greater than the CO_2_ extracted.

Such a formidable challenge necessitates the formulation and implementation of effective strategies. The CO_2_ management technologies employ a range of advanced materials, both natural and engineered, selected for their capacity to effectively adsorb or react with CO_2_ under pragmatic conditions. Key materials, including zeolites, amine-based sorbents, activated carbon, ionic liquids, biochar, alkaline earth metal oxides, and metal-organic frameworks (MOFs), have been tested for CO_2_ capture at variable scales. Zeolites, crystalline aluminosilicates with microporous frameworks with modular porosity and thermal stability, have been widely utilized for post-combustion CO_2_ capture, albeit with a limited capture capacity.^34^ Amine-based sorbents, both in liquid and solid-supported varieties, chemically bind CO_2_ via carbamate formation, providing high CO_2_ capture efficiency. Although issues like oxidative degradation and the requirement of significant regeneration energy for CO_2_ removal have seriously hampered their practical applications.^35^ Activated carbon, often derived from biomass, is cost-effective and highly porous, suitable for physisorption under various conditions. However, this benign material offers a low CO_2_ capture and conversion potential to meet the industrial requirements.^36^ Similarly, ionic liquids, organic salts in the liquid state, enable reversible CO_2_ capture with low volatility and high thermal stability, making them attractive for integration into hybrid systems. Interestingly, the low stability and high cost associated with ionic liquids pose serious questions about their large-scale implementations.^37^^,^^38^ Biochar, produced from pyrolyzed organic waste, not only captures CO_2_ through surface adsorption but also enhances soil carbon sequestration when applied agriculturally. However, the manufacturing process of biochar leaves a significant carbon footprint, which jeopardizes the overall carbon balance.^39^^,^^40^ Alkaline earth metal oxides, notably CaO, are widely used in mineral looping processes, where CO_2_ is converted into stable CaCO_3_. This captured CO_2_ can be released back from the mineral following calcination, enabling purified CO_2_ generation via a cyclical operation in industrial setups.^41^ MOFs, characterized by high surface area and tuneable pore structures, offer exceptional CO_2_ uptake and selectivity, though challenges remain in terms of their scalability, cost viability, and moisture sensitivity.^42^

In addition to the engineered materials, natural and industrially derived silicates such as basalt,^43^ olivine,^44^ steel slag,^45^ and fly ash^46^ serve as abundant sources of reactive group-II divalent cations (Ca^2+^, Mg^2+^) for permanent mineral carbonation. Treatment methods include grinding to increase surface area, thermal activation (especially for CaO and silicates), acid or base leaching to extract reactive species, and chemical modification to improve selectivity and kinetics. Moreover, material performance is governed by factors, such as porosity, chemical stability, regeneration energy, and CO_2_ selectivity, all of which influence deployment at scale. Combining these materials strategically, e.g., using MOFs or amines for initial capture and CaO or silicates for long-term storage, can enhance overall system efficiency, reduce environmental impact, and support scalable CO_2_ sequestration pathways in alignment with climate goals. Beyond efficiency, carbon management technologies must also be practical and sustainable. Materials used for CCU and CDR purposes should be affordable for widespread adoption, especially in developing regions. Scalability is essential so that these solutions can be deployed at industrial levels without major resource or energy constraints. Environmental safety is equally important, meaning materials and processes should have minimal ecological impact, low toxicity, and low energy needs for operation. Using waste-derived or naturally abundant resources like biochar and steel slag can help lower both costs and emissions, while ensuring a circular economy.

Nevertheless, any CO_2_ mitigation technological advancement followed by CO_2_ utilization to be regarded as viable, it is essential that it yields products with intrinsic value that can adequately offset the considerable costs associated with CO_2_ capture and conversion processes. Moreover, these products must address extensive and varied markets, thereby ensuring both scalability and widespread acceptance. This dimension is particularly crucial, considering that CO_2_-derived products are projected to be manufactured at volumes surpassing several million tons, thereby requiring the presence of a market capable of accommodating such substantial quantities without disturbing the balance between supply and demand.

In the context of India, the construction and food sectors present significant opportunities for CO_2_ utilization. India’s rapidly growing economy and expanding population drive demand in these sectors, offering a promising avenue for integrating CO_2_-derived products. The construction sector, characterized by its escalating demand for raw materials including cement and aggregates, alongside the food industry, which is dependent on fertilizers and various agricultural inputs, is optimally positioned to incorporate significant volumes of CO_2_-derived products. It was estimated that India’s construction aggregate market is expected to grow from 36.32 billion USD in 2024 to 81.65 billion USD by 2035. On the other hand, India’s fertilizer market size was valued at around USD 10.8 billion in 2024 and is projected to reach USD 14 billion by 2030.^47^^,^^48^ Therefore, focusing on these sectors, India can efficiently manage the supply-demand equilibrium while leveraging the environmental and economic benefits of CO_2_ utilization.

The successful implementation of CO_2_ utilization strategies in India depends on addressing three critical factors. Initially, the terminal outputs obtained from the capture and conversion of CO_2_ must constitute high-value commodities that possess the potential to yield adequate economic returns, thereby rationalizing the associated investment. Examples include advanced construction materials and fertilizers that not only meet industry requirements but also contribute to sustainability goals. Second, these products must cater to substantial markets with extensive applications across multiple industries, ensuring their scalability and consistent demand. Ultimately, the production of these commodities utilizing the presently employed conventional techniques generates substantial quantities of CO_2_, thereby making a significant contribution to anthropogenic greenhouse gas emissions. The incorporation of a CCU strategy fundamentally modifies this trajectory by capturing CO_2_ emissions at their point of origin and transforming them into valuable products. This methodology not only diminishes the direct emission of CO_2_ into the atmosphere but also establishes a regenerative cycle wherein carbon is reused instead of being discarded. By converting CO_2_ into fuels, chemicals, construction materials, or polymers, CCU facilitates a circular carbon economy, where carbon atoms are perpetually cycled through industrial processes rather than being regarded as waste. This circularity reduces dependence on virgin fossil carbon sources and alleviates the environmental impact of manufacturing. Moreover, it aligns with sustainable development objectives by nurturing resource efficiency, lessening reliance on non-renewable inputs, and bolstering the resilience of industrial supply chains. The implementation of CCU technologies thus signifies a transition from a linear “take-make-dispose” model to a closed-loop system, promoting decarbonization while simultaneously generating economic value from captured emissions.

This review will specifically examine two prominent CO_2_ utilization technologies: mineralization and fertilizer production. Mineralization presents a significant opportunity to convert carbon dioxide into stable, solidified compounds that are appropriate for incorporation into construction materials, whereas the production of fertilizers utilizes sequestered CO_2_ to augment agricultural efficiency. Both approaches align with the dual objectives of generating high-value products and addressing the extensive market requirements essential for the large-scale deployment of CO_2_ utilization technologies. Through the emphasis on these technological advancements, India has the potential to make considerable progress in attaining its climate objectives while simultaneously preserving economic development.

### CO_2_ mineralization: An integral approach for carbon sequestration and utilization

**Figure 3:**
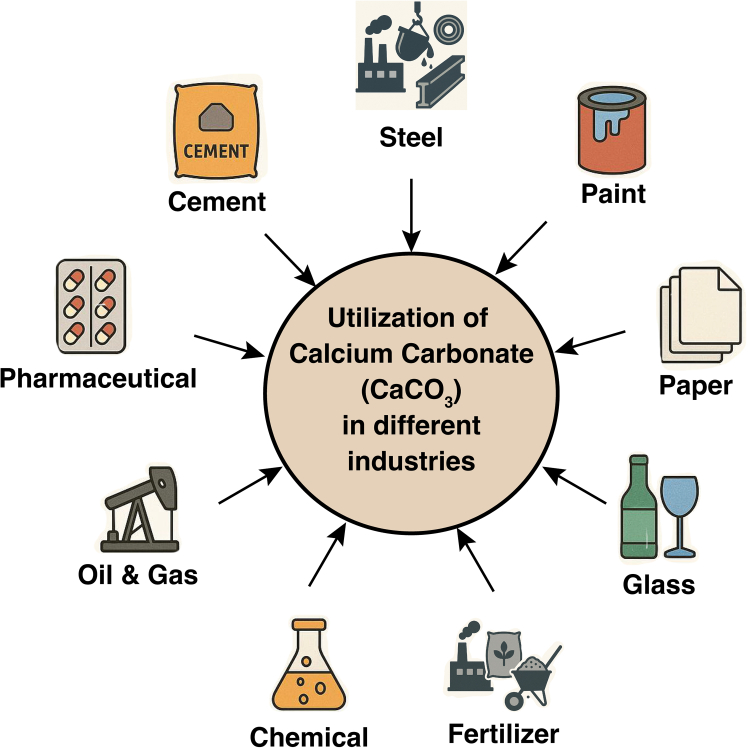
Variable applications of calcium carbonate in industry

The mineralization of CO_2_ constitutes a pivotal mechanism that has significantly influenced the regulation of atmospheric CO_2_ levels throughout geological epochs. This process encompasses the engagement of CO_2_ with group II divalent cations, including calcium (Ca^2+^) and magnesium (Mg^2+^), resulting in the production of stable water-insoluble carbonate minerals, which has diverse applications in industries (Figure 3).^49^ In the domain of CCUS, mineralization is regarded as the most reliable and enduring mechanism for the sequestration of CO_2_. Although CO_2_ mineralization is frequently linked to geological storage within basalt, sandstone, and shale formations, it can be systematically classified into *in-situ* and *ex situ* mineralization contingent upon the spatial context of the reaction.^50^

*In situ* mineralization involves the injection of CO_2_ into underground strata composed of reactive lithologies, including basalt or volcanogenic sandstone, characterized by an abundance of divalent cations. The injected CO_2_ reacts with the rock minerals, forming stable carbonates. This approach leverages natural geological formations to securely store CO_2_ underground. Conversely, *ex situ* mineralization entails carbonation processes that occur above the surface, utilizing reactive feedstocks like fly ash or industrial by-products that are rich in divalent cations.^51^

*ex situ* mineralization can be further subdivided into direct and indirect methods. In the direct method, CO_2_ is mineralized in a single step through a reaction with alkaline substances to form carbonate solids. However, this process is chemically complex and entropically unfavorable, requiring considerable energy inputs. Conversely, the indirect methodology encompasses a bifurcated procedure: initially, reactive divalent cations (Ca^2+^ and Mg^2+^) are obtained from feedstocks or mineral matrices, followed by the reaction of these cations with bicarbonate ions produced from CO_2_ to yield stable carbonate compounds. This indirect approach, while more elaborate, offers a scalable pathway for removing gigatons of CO_2_ annually, aligning with global climate goals, such as achieving net-zero emissions.^52^

#### Biological perspectives on CO_2_ mineralization

Although the process of CO_2_ mineralization has been historically recognized to transpire over extensive geological timescales encompassing millions of years, it is important to note that biological systems have ingeniously evolved various mechanisms to significantly expedite this naturally sluggish process. One notable significant mechanism that has been elucidated is the enzymatic hydrolysis of CO_2_, which is primarily enabled by the presence of carbonic anhydrase (CA), a metalloenzyme characterized by the inclusion of zinc as an essential element of its composition.^53^ The importance of CA is critical, as it proficiently facilitates the interconversion between carbon dioxide and bicarbonate ions (HCO_3_^−^) in aqueous systems, a biochemical process that is vital for photosynthesis and energy transformation across a diverse range of organisms, particularly in photosynthetic flora and specific microorganisms.^54^

Under standard environmental conditions, CO_2_ possesses the ability to dissolve in aqueous solutions, resulting in the generation of bicarbonate ions; however, it is imperative to recognize that this reaction is fundamentally thermodynamically unfavorable, leading to a considerably sluggish reaction rate that can obstruct natural carbon cycling.^55^ The catalytic proficiency of CA markedly enhances the reaction kinetics by an astounding magnitude of as much as one million times per second,^56^^,^^57^ thereby positioning it as an extraordinarily effective natural catalyst that performs an essential function in the global carbon cycle.^58^ Once the bicarbonate ions are generated, they readily engage in subsequent reactions with divalent cations, such as calcium or magnesium, to yield a stable carbonate salts, which ultimately completes the vital mineralization process that sequesters carbon in solid forms.

This enzymatic pathway, characterized by its remarkable efficiency, offers two significant advantages that are highly relevant in the context of contemporary environmental challenges: it not only drastically reduces the timescale associated with CO_2_ mineralization, thereby rendering the process feasible within practical and manageable timescales, but it also effectively lowers the energy requirements necessary for carbonation reactions. Consequently, this reduction in energy demands serves to diminish both the operational costs associated with such processes and the overall carbon footprint, making these mechanisms increasingly attractive for applications in carbon capture and storage technologies that aim to mitigate climate change impacts.

#### Challenges in industrial applications of enzymatic carbonation

Despite the significant potential that enzymatic carbonation presents for a range of industrial uses, the actual deployment of this technology is fraught with numerous substantial obstacles that require resolution.^56^ CA enzymes, which are remarkably intricate biological catalysts comprised of meticulously folded polypeptide chains consisting of diverse amino acids that are systematically organized in a three-dimensional structure, demonstrate their peak catalytic effectiveness and functionality when exposed to specific physiological conditions that are commonly characterized by a pH level that approximates the neutral point of around 7.0, a temperature sustained at roughly 30°C, which is essential for optimal enzymatic activity, and a pressure that typically hovers around 1 atm, thus developing an environment that is favorable for their biochemical interactions.^59^ However, these ideal conditions are exceedingly difficult to reproduce within the context of industrial operations, which frequently entail elevated temperatures, increased pressures, and the presence of corrosive acidic flue gases. Consequently, the CA enzymes exhibit a significant propensity for denaturation, wherein they undergo structural changes that result in a marked decrease in their catalytic efficiency when exposed to such extreme and harsh environmental conditions.^56^

Furthermore, the intricate nature of the synthesis process for CA or similar enzymes is inherently complex and entails considerable financial investment, thereby introducing an additional layer of difficulty to the scenario. These pivotal factors, encompassing thermal instability, increased susceptibility to acidic conditions, and the considerable costs associated with enzyme production, ultimately result in the impracticality and economic infeasibility of employing enzymatic carbonation for extensive industrial purposes, especially within the framework of directly capturing carbon dioxide from flue gas emissions, which represent a major contributor to greenhouse gas emissions.

#### Synthetic catalysts derived from carbonic anhydrase

In an effort to transcend the intrinsic limitations associated with enzymatic carbonation, an increasing number of scholars are assiduously directing their research endeavors toward the pioneering formulation of synthetic catalysts that are specifically engineered to closely replicate the catalytic functionalities demonstrated by CA. The principal aim of these synthetic catalysts is to proficiently emulate the extraordinary efficiency and operational characteristics that are characteristic of natural CA, while concurrently ensuring that these alternatives exhibit improved durability, enhanced scalability, and a more advantageous cost-effectiveness profile. Additionally, these synthetic alternatives are scrupulously designed with the objective of withstanding the stringent and often arduous conditions that are conventionally found in various industrial applications, which frequently involve elevated levels of pollutants, such as sulphur oxides (SO_x_) and nitrogen oxides (NO_x_).^60^

Significant advancements have been reported from startups focusing on CCUS, wherein synthetic catalysts modeled after enzymes are undergoing evaluation at pilot scales.^60^ These catalysts have exhibited the capability to promote effective CO_2_ carbonation within industrial environments, positioning them as a credible substitute for biological enzymes. The emergence of enzyme-inspired synthetic catalysts represents a crucial advancement in the domain of CO_2_ mineralization. These catalytic agents facilitate not only the effective sequestration of CO_2_ from ambient air or flue gas emissions but also act as a precursor for the later application of CO_2_ in the synthesis of carbonate minerals. Such advancements possess significant potential to meet global climate objectives by providing scalable and economically viable carbon capture and storage methodologies (Table 1).

**Table 1:** CO_2_ capture by carbonic anhydrase enzymes and chemical mimics

Type	Name/Strain/Compound	Mechanism	CO_2_ Capture Rate	Efficiency/Notes	Estimated Capture (tons)
Enzyme	Human Carbonic Anhydrase II (HCA II)^61^	Zn^2+^ metalloenzyme catalyzing CO_2_ + H_2_O ⇌ HCO_3_^−^ + H^+^	Up to 10^6^ CO_2_ molecules/sec	Extremely high turnover rate; lab-scale only	<0.01 tons (lab use)
Enzyme	Thermophilic CA from Sulfurihydrogenibium azorense^62^	High-temperature stability, catalyzes CO_2_ hydration	Active at >70°C	Suited for industrial flue gas; immobilization possible	∼0.1–1 tons/year (pilot scale)
Enzyme	Recombinant CA expressed in E. coli ^63^	Engineered for expression yield and activity	Depends on expression system	Useful for immobilization in membranes or columns	Pilot scale: <1 tonne/year
Chemical Mimic	Zinc(II)-cyclen complex^64^	Mimics CA active site with Zn^2+^ and N-donor ligands	Catalytic activity ∼10^4^–10^5^ s^−1^	Used in aqueous capture solutions	Lab scale: <0.01 tons
Chemical Mimic	Cobalt-based Schiff base complex^65^	Redox-active CO_2_ hydration mimic	Variable activity	Tested in flue gas simulants	<1 tonne (research stage)
Chemical Mimic	Ionic liquids with CA-like activity^66^	Physico-chemical absorption + catalytic sites	∼0.5–1 mol CO2/mol IL	Some scale-up potential	1–5 tons/year (pilot trials)
Chemical Mimic	Zinc(II)-cryptand complex^60^	Mimics CA active site	8.82 ± 0.98 × 10^−3^ s^−1^	Used in aqueous capture solutions, including flue gas samples	0.1 tons/year (pilot trial)
Enzyme–Chemical Hybrid	CA immobilized on silica or polymer supports^67^	Enhances durability and recyclability	Maintains high activity over cycles	Potential for modular deployment	Pilot scale: 1–10 tons/year

Furthermore, synthetic catalysts exhibit the capability to interlink the mechanisms of CO_2_ capture with its subsequent utilization, thereby promoting the transformation of sequestered CO_2_ into economically advantageous products. This confluence of CO_2_ capture and utilization/mineralization is congruent with the overarching objectives of CCUS, thereby facilitating the global transition toward a low-carbon economy.

In conclusion, although biological enzymatic mechanisms provide significant insights into the acceleration of CO_2_ mineralization, their applicability at an industrial scale remains hindered by prevailing limitations. Synthetic catalysts designed to emulate natural enzymes present a compelling alternative, integrating the efficacy of biological processes with the robustness required for industrial applications. As investigations in this domain advance, enzyme-inspired catalytic methodologies are anticipated to assume a critical role in the enhancement of CO_2_ mineralization technologies, propelling the global community closer to realizing its aspirations for carbon neutrality.

### CO_2_ utilization for fertilizer production

The worldwide population is anticipated to rise by roughly 35% in the ensuing forty years, thereby exerting considerable strain on the agricultural industry to augment food output while navigating the limitations imposed by finite arable land.^68^ As the primary source of food, agriculture must rely increasingly on improving crop yields rather than expanding farmland.^69^ In the context of contemporary agricultural practices and with the ever-increasing global population necessitating enhanced food production methodologies, it becomes abundantly clear that fertilizers play an indispensable role in ensuring global food security, given that they exert a direct and profound influence on both the growth and productivity of various plant species, thereby facilitating the overall efficiency and sustainability of food systems worldwide.

Throughout history, the application of fertilizers can be traced to primitive agricultural methodologies in the Neolithic era, wherein organic substances like animal waste and mineral supplements were utilized to improve soil nutrient content.^70^ The introduction of synthetic fertilizers in the early 20th century represented a significant transformation in worldwide agricultural efficiency. In particular, the development of the Haber-Bosch process in 1909, enabling the industrial fixation of atmospheric nitrogen (N_2_) into ammonia (NH_3_), revolutionized nitrogen fertilizer production and catalyzed what is often referred to as the Green Revolution.^71^ Today, nitrogen-based synthetic fertilizers contribute substantially to crop yield increases and are considered essential to feeding the modern global population. It is approximated that nearly one-third of the global food supply is reliant on synthetic fertilizers generated through the Haber-Bosch process, which continues to be the preeminent technique for ammonia synthesis, representing over 96% of worldwide production.^71^

In addition to their role in enhancing agricultural output, fertilizers intersect significantly with the global carbon cycle. Through increased photosynthetic activity, fertilized crops can enhance the uptake of atmospheric CO_2_ and contribute to short-term carbon sequestration in plant biomass and soil organic matter.^72^ Nevertheless, the production methodologies linked to synthetic fertilizers, particularly those that are primarily nitrogen-centric, exhibit an exceedingly elevated requirement for energy. This manufacturing process is currently heavily dependent on fossil fuel sources, which, as a result, contributes to the emission of substantial quantities of carbon dioxide, adversely affecting the ecosystem.^73^^,^^74^ Furthermore, the utilization of fertilizers can lead to the release of nitrous oxide (N_2_O), a greenhouse gas that possesses a global warming potential approximately 298 times that of CO_2_ over a centennial period.

Efforts to integrate CO_2_ utilization into fertilizer production present a promising avenue for mitigating these environmental impacts. Technologies such as green ammonia synthesis using renewable hydrogen, CO_2_-derived urea, and carbon-enriched biofertilizers offer potential pathways to decouple fertilizer production from fossil fuel dependence while enhancing carbon efficiency. Such innovations are critical not only for reducing the carbon footprint of agriculture but also for transitioning toward more circular and sustainable nutrient management systems.

#### Environmental implications of synthetic fertilizers and organic fertilizer

**Figure 4:**
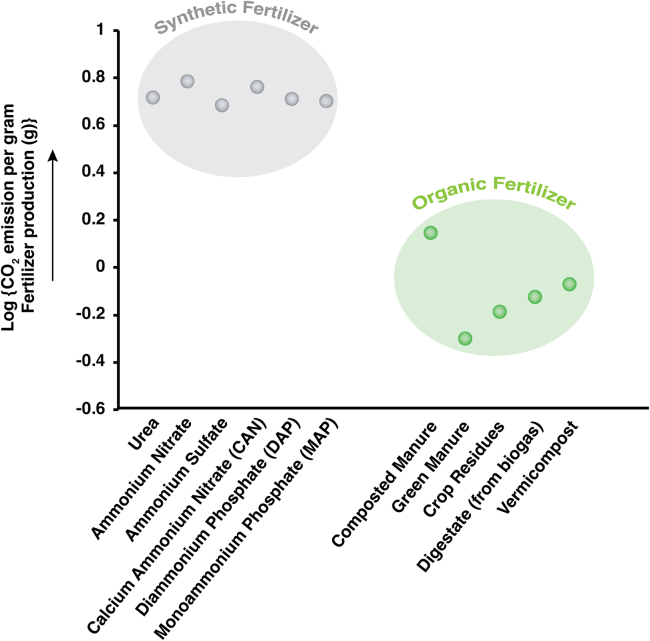
Comparative CO2 emission for variable fertilizer usage Comparison of CO_2_ emissions per gram of production for synthetic and organic fertilizers, showing higher emissions for synthetic types and lower emissions for organic alternatives, presented on a logarithmic scale.^76^

While synthetic fertilizers have markedly enhanced agricultural yields, their application incurs significant environmental ramifications. The energy-intensive Haber-Bosch process is fundamental to the fabrication of synthetic fertilizers, which predominantly depends on fossil fuels and necessitates approximately 40–60 GJ per ton of ammonia produced.^75^^,^^76^ This reliance results in considerable emissions of greenhouse gases, including CO_2_. Furthermore, the excessive application of nitrogen-rich chemical fertilizers has led to the accumulation of reactive nitrogen within ecosystems, engendering detrimental consequences, such as soil degradation, water contamination, and atmospheric pollution (Figure 4).

In order to effectively address and alleviate the numerous environmental challenges that are currently confronting our agricultural practices, there has been a notable and significant resurgence in the adoption and utilization of organic fertilizers, which encompass a diverse array of materials, such as animal manure, composted organic waste, sewage sludge, by-products from food processing, and various forms of municipal biosolids. These organic fertilizers are widely regarded as environmentally benign alternatives to conventional chemical fertilizers and are recognized for their potential contributions to the promotion of sustainable agricultural systems that prioritize ecological balance and resource conservation.^77^ Nevertheless, it is imperative to acknowledge that the application and utilization of organic fertilizers are not devoid of certain limitations and inherent challenges. In general, it is observed that organic fertilizers, which are derived from natural sources and are often characterized by their environmentally friendly composition, tend to contain a comparatively diminished concentration of vital nutrients that are crucial for plant growth and development, and furthermore, these organic options also demonstrate a more gradual and prolonged rate of nutrient release into the soil environment when juxtaposed with their synthetic counterparts, which are chemically formulated to deliver nutrients rapidly and in higher concentrations, thereby necessitating the application of significantly larger volumes of organic fertilizers in order to ensure that crops receive effective, timely, and adequate nutrition that meets their physiological requirements for optimal growth and yield.^78^ This requirement for increased volume of application consequently renders organic fertilizers relatively more costly in comparison to traditional chemical options.^79^ Moreover, it is crucial to consider that the prolonged and excessive application of organic fertilizers can result in the accumulation of salts, nutrients, and potentially harmful heavy metals within the soil matrix, which can have detrimental impacts on plant growth, the health of soil organisms, the quality of water resources, and ultimately, human health, and well-being.

Calculating the total carbon footprint of fertilizer use requires a life cycle approach that incorporates all major CO_2_ and CO_2_-equivalent (CO_2_-eq) emission sources. Manufacturing emissions are typically the largest contributor, especially for synthetic nitrogen fertilizers produced via the energy-intensive Haber-Bosch process, which relies heavily on fossil fuels and generates substantial process-related CO_2_. Transportation emissions arise from moving fertilizers from plants to farms, with greater proportional impacts for low-nutrient-density products like organic manures and composts. Soil-related emissions include direct CO_2_ release from carbon-containing fertilizers and nitrous oxide (N_2_O) from nitrification-denitrification, as well as indirect emissions from nitrogen losses via volatilization and leaching, which generate N_2_O downstream. Across the life cycle, synthetic nitrogen fertilizers generally emit about 4.5–6.3 g CO_2_-eq per gram of nutrient applied, with manufacturing alone accounting for 70%–80% of this total.^76^ In contrast, organic fertilizers, such as composted manure or crop residues, emit 0.4–1.6 g CO_2_-eq per gram of nutrient, reflecting lower energy inputs and slower nutrient release, although methane emissions from poorly managed composting can increase their footprint.^76^ A complete accounting of these sources allows for more accurate comparisons between fertilizer types and supports the development of lower-carbon nutrient management strategies (Figure 4).

#### Biofertilizers as a sustainable alternative

Given the apparent limitations and disadvantages associated with the traditional application of both synthetic and organic fertilizers, which frequently pose considerable threats to environmental integrity and soil vitality, biofertilizers have surfaced as a notably more sustainable and ecologically responsible alternative that corresponds with contemporary agricultural methodologies focused on sustainability.^80^ Biofertilizers are composed of either active or dormant microbial entities that function synergistically to enhance soil fertility via multiple mechanisms, including the fixation of atmospheric nitrogen, the solubilization of essential phosphates, the production of advantageous plant growth regulators, and the suppression of various soil-borne pathogens and diseases that could adversely affect crop vitality.^81^ The utilization of biofertilizers within agronomic frameworks has been empirically substantiated to yield significant enhancements in crop productivity, generally fluctuating between 20% and 30%.^82^ This offers a feasible and efficacious strategy for augmenting agricultural output while simultaneously safeguarding and conserving the adjacent environmental ecosystems. Therefore, the incorporation of biofertilizers into agricultural methodologies signifies an advanced movement toward realizing sustainable agricultural progress, which not only bolsters food security but also promotes ecological equilibrium and fortitude within agro-ecosystems.

Carbon footprint assessment of microorganism-based biofertilizers involves life cycle assessment (LCA) that accounts for emissions from microbial cultivation, carrier preparation, formulation, packaging, transport, and field application. Common examples include nitrogen-fixers (*Rhizobium*, *Azotobacter*, *Azospirillum*), phosphate-solubilizers (*Bacillus*, *Pseudomonas*), potassium-mobilizers (*Bacillus mucilaginosus*), arbuscular mycorrhizal fungi (*Rhizophagus* spp.), and cyanobacteria (*Anabaena*, *Nostoc*). Compared with synthetic fertilizers, these products often have significantly lower emissions due to lower energy intensity and smaller application rates. However, precise quantification remains challenging because production methods, carrier materials, energy sources, and application conditions vary widely. Soil-related indirect emissions, such as nitrous oxide release during nutrient cycling, are also difficult to measure and may fluctuate by site and crop type. Moreover, nutrient content in microbial formulations is not standardized, complicating cross-study comparisons. Despite these uncertainties, microorganism-based biofertilizers present clear environmental advantages, offering a lower-carbon pathway for enhancing soil fertility while reducing reliance on synthetic inputs. Improving field-level emission data and developing standardized calculation frameworks will further validate their role in sustainable agriculture and help integrate them into climate-smart nutrient management strategies.

#### Striking a balance for sustainable agriculture

The attainment of sustainable agricultural practices necessitates a carefully considered equilibrium that harmonizes the principles of effective plant nutrition management with the imperative of safeguarding environmental integrity.^83^ It is essential to recognize that every classification of fertilizer—comprising synthetic fertilizers, organic enhancements, and biofertilizers—demonstrates distinct advantages and drawbacks when assessed regarding nutrient provision, improvement of soil health, and the facilitation of optimal agricultural productivity. Consequently, the implementation of an integrated nutrient management strategy that synergistically incorporates various types of fertilizers can significantly enhance agricultural productivity while concurrently reducing adverse environmental consequences. Through the implementation of a comprehensive strategy, participants in the agricultural domain can strive toward the attainment of a sustainable future that not only satisfies the requirements of food production but also safeguards the integrity of our ecosystems for future generations.

Nonetheless, despite employing such a comprehensive methodology, a considerable carbon footprint is still linked to each of the three categories of fertilizers. Tackling this challenge requires the advancement of technologies capable of harnessing CO_2_ emissions within agricultural frameworks, thus progressing toward carbon neutrality.

#### CO_2_ utilization for carbon-negative fertilizer production

In the pursuit of establishing a carbon-neutral agricultural paradigm, scholars are investigating the viability of CCU technologies for the integration of CO_2_ into the synthesis of fertilizers.^84^ One promising approach involves the utilization of captured CO_2_ in the synthesis of urea. Another approach can be the integration of green ammonia, produced using renewable energy sources, into fertilizers, becoming a possibility to close the carbon loop within agriculture. This innovation would transform fertilizer production into a carbon-negative process, significantly reducing the overall environmental impact of agriculture.^85^

In the Indian context, where agriculture is a cornerstone of the economy and food security, the implementation of CCU technology holds immense potential. A system that utilizes CO_2_ emissions to produce fertilizers could mitigate the adverse environmental effects of traditional practices while enhancing agricultural sustainability. Researchers are not only focusing on developing combination fertilizers but are also working toward establishing a carbon-negative fertilization system tailored to the unique needs of Indian agriculture.

The global challenge of feeding a rapidly growing population necessitates innovations that increase agricultural productivity while minimizing environmental harm. Fertilizers, whether synthetic, organic, or biofertilizers, have played a pivotal role in meeting this challenge, yet each comes with inherent limitations and environmental costs. To address these issues, integrating CCU technologies into fertilizer production offers a transformative solution. By utilizing captured CO_2_ in synthesizing urea and simultaneously incorporating green ammonia, the agriculture sector can achieve a sustainable and carbon-neutral trajectory.

This approach not only responds to the urgent demand for sustainable agricultural practices but also corresponds with overarching international objectives aimed at diminishing greenhouse gas emissions and addressing climate change. Through ongoing research and technological innovations, the implementation of CO_2_ in fertilizer manufacturing possesses the capacity to transform agricultural methodologies, safeguarding food security for forthcoming generations while maintaining the ecological integrity of the planet.^86^

## Designing a comprehensive template for CO_2_ utilization in India

The climate action strategy that has been articulated by the Indian government, commonly referred to as the five nectar elements or Panchamrit,^29^ encompasses a comprehensive array of ambitious objectives that are aimed at addressing the pressing challenges posed by climate change, which include the following key initiatives.

1. The establishment of a substantial non-fossil energy capacity, specifically targeting the achievement of 500 GW by the year 2030, representing a significant shift away from reliance on fossil fuels, which has long been a cornerstone of energy production.^29^
2. A commitment to sourcing an impressive 50% of the nation’s total energy requirements from renewable energy sources by the year 2030, thereby enhancing energy security while simultaneously fostering sustainable development practices.^29^
3. An ambitiouSs goal to reduce the total projected carbon emissions by a staggering one billion tons between the current date and 2030, which reflects a proactive approach to mitigating the detrimental impacts of greenhouse gas emissions on the environment.^29^
4. A targeted reduction in the carbon intensity of the Indian economy by a remarkable 45% by the year 2030, utilizing 2005 levels as a baseline, which illustrates the government’s determination to transition toward a more sustainable economic framework.^29^
5. An overarching aim to accomplish the significant milestone of achieving net-zero emissions by the year 2070, which underscores India’s long-term commitment to combating climate change on a global scale.^29^

The Prime Minister of India has publicly committed to these aforementioned climate objectives during the COP26 held in the year 2021, as documented by the Ministry of External Affairs (2021). Following this pivotal moment, India proceeded to submit its inaugural Long-term Strategy for Low Carbon Development (LT-LEDS)^87^ during the subsequent COP27, which took place the following year. Given that a considerable 80% of India’s carbon dioxide emissions are attributed to industries that are particularly challenging to decarbonize, the LT-LEDS delineates specific strategies aimed at the decarbonization of various critical sectors, including power generation, industrial operations, transportation, building infrastructure, and urban development. Nevertheless, it is important to note that the LT-LEDS falls short in providing sufficiently explicit policy directives that articulate the government’s approach to achieving net-zero emissions beyond the scope of its existing policies and programs. Despite this constraint, the Niti Aayog, recognized as India’s foremost policy research institution, unveiled an extensive strategic framework for CCUS in November 2022. This particular report meticulously examines the potential pathways and requisite policies essential for the realization of the ambitious Panchamrit goals, thereby contributing to a more sustainable future for India in the context of global climate action.

### Decarbonizing India’s power sector

Coal remains the principal source of energy within the extensive energy framework of India, accounting for a substantial 73% of the country’s overall energy composition, while concurrently producing nearly 75% of the total electricity utilized throughout the nation. Despite the ambitious aspirations articulated within the Panchamrit framework, which establishes a target of achieving an impressive 500 GW of non-fossil energy capacity by the year 2030, the anticipated increase in India’s power capacity to a substantial 900 GW highlights the ongoing and substantial reliance on coal-based energy solutions. The ongoing reliance on coal can be ascribed not solely to its plentiful presence within the nation’s spatial boundaries but also to its exceptional energy density, dependability, and responsiveness, which are in significant opposition to the more sporadic and less foreseeable characteristics of renewable energy alternatives.^88^

The CO_2_ emissions tied to electricity generation are heavily shaped by the kind of fossil fuel used, with coal emitting about 0.9–1.0 kg CO_2_ for every kilowatt-hour, petroleum products contributing roughly 0.7–0.8 kg CO_2_ per kilowatt-hour, and natural gas producing around 0.4–0.5 kg CO_2_ per kilowatt-hour. In the context of India, where coal constitutes nearly 70% of the energy mix, this results in an annual emission of over 1.1 gigatonnes of CO_2_, specifically from the electricity sector.^89^ The challenge of diminishing this carbon footprint while ensuring energy security necessitates the extensive implementation of CCU and CDR technologies.

The implementation of CCU technologies, like harnessing captured CO_2_ for boosting oil recovery, producing chemical precursors, or crafting construction materials, holds the promise of reducing net emissions and unlocking economic opportunities. For instance, the process of mineral carbonation, utilizing industrial by-products like steel slag or fly ash, could sequester millions of tonnes of CO_2_ each year in India. CDR strategies, including bioenergy combined with carbon capture and storage (BECCS), direct air capture (DAC), and afforestation, can actively extract CO_2_ from the atmosphere, although DAC remains prohibitively expensive (exceeding $100 per tonne of CO_2_) relative to point-source capture from coal facilities (approximately $40–$50 per tonne of CO_2_ in India).

Achieving energy security while pursuing decarbonization will necessitate a diversification of the energy portfolio toward renewable sources, aiming for 500 GW of non-fossil fuel capacity by 2030, enhancing grid efficiency to minimize transmission losses (currently around 18%), and upgrading coal-fired power plants to supercritical or ultra-supercritical technologies to decrease emission intensity. Strategic investments in CO_2_ transportation and storage infrastructure, regional CCU centers, and industrial symbiosis networks can further optimize carbon management efforts.

The thorough integration of CCU and CDR technologies within the operational framework of the energy sector represents a notably pragmatic and strategically astute response to this pressing challenge. By facilitating the effective capture and subsequent sequestration of carbon dioxide emissions produced by coal-fired power plants, CCU and CDR have the potential to significantly mitigate the adverse environmental impacts associated with fossil fuel consumption, all while circumventing the requirement for an abrupt and complete transition away from coal as a predominant energy source. This specific strategy not only guarantees a balanced and progressive transition toward a more sustainable energy system but also aligns seamlessly with India’s essential decarbonization objectives established for the year 2030, thereby promoting a more responsible and environmentally-conscious energy policy.

### Hydrogen as a decarbonization pathway

In addition to coal, the Indian administration has recognized hydrogen as an essential energy vector for realizing its decarbonization goals. The focus is primarily on green hydrogen, which is produced via electrolysis powered by renewable energy. Green hydrogen represents a cornerstone of sustainable energy systems, yet its widespread adoption faces significant hurdles, including.

1. The limited availability of pure deionized water for electrolysis.
2. Challenges in scaling up efficient and cost-effective catalytic systems.^90^

As a result, large-scale green hydrogen production remains in its nascent stages in India. Conversely, Indian industries have long relied on non-renewable hydrogen in sectors, such as syngas production, methane synthesis, and ammonia manufacturing.^91^ CCUS technologies present an opportunity to transform this non-renewable hydrogen into blue hydrogen, wherein CO_2_ emissions are captured and stored during production.^92^^,^^93^^,^^94^^,^^95^^,^^96^

The International Energy Agency (IEA) has conducted comprehensive analyses and forecasts, projecting that by the year 2030, an impressive 50% of the total global demand for hydrogen will need to be satisfied through sources that emit low levels of carbon, which will notably include a significant composition of two-thirds derived from what is categorized as green hydrogen, while the remaining one-third will be sourced from blue hydrogen, thereby reflecting a crucial transition toward more sustainable energy practices.^97^ Deploying CCUS in India’s non-renewable hydrogen industries can facilitate this transition, reducing emissions in hydrogen-derived industries and enabling India to meet its decarbonization targets effectively.

### Developing a robust CCUS policy framework

In order to meet its climate commitments under the Paris Agreement and achieve long-term carbon neutrality goals, India must establish a comprehensive and pragmatic CCUS policy framework. This framework ought to explicitly target the hard-to-abate industrial sectors that are particularly challenging to achieve decarbonization, specifically, electricity generation, steel production, cement manufacturing, petrochemical operations, and chemical sectors. These sectors are not only critical for the advancement of India’s economic architecture but together account for over 80% of the nation’s overall CO_2_ emissions. Given the scale and intensity of emissions from these industries, the implementation of CCUS technologies is not just an option but a necessity for India’s low-carbon transition.

In the global context, countries have adopted a range of policy mechanisms to support decarbonization. These instruments broadly fall into two categories.

1. Compulsory mechanisms: These mechanisms are designed to enforce stringent and mandatory GHG reduction targets through an array of policies, regulations, and compliance requirements that industries must adhere to in order to contribute to national and global climate objectives.^98^
2. Voluntary mechanisms: These mechanisms serve to incentivize industries to actively offset their emissions by engaging in the purchase of carbon credits from projects that are dedicated to significantly reducing or entirely removing GHG emissions from the atmosphere.^98^

**Figure 5:**
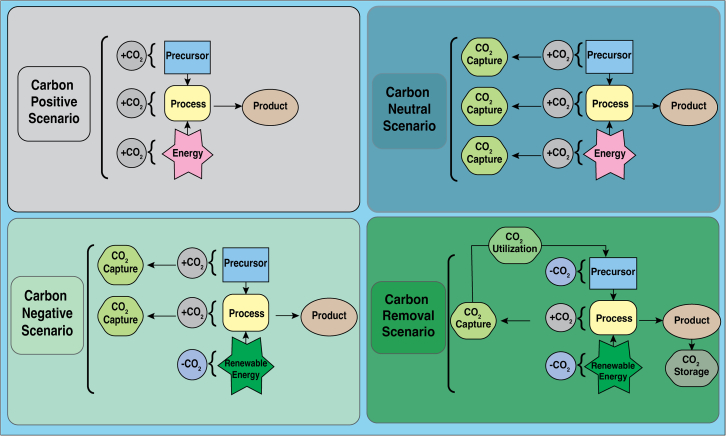
The different carbon management scenarios for sustainable industrial operations

An effective national CCUS policy should aim to balance both these mechanisms. While compulsory approaches create the necessary enforcement for industrial compliance, voluntary mechanisms provide the incentive for industries to innovate, invest, and adopt technologies like CCUS. This dual approach guarantees that the mitigation of emissions is not only implementable but also financially attractive to interested parties. A notably beneficial feature of CCUS resides in its ability to yield products of significant economic value. Through the process of sequestering carbon dioxide and transforming it into economically beneficial products, such as chemical substances, sustainable energy sources, building materials, and nutrient enhancers, CCUS presents a dual opportunity for revenue generation for industrial sectors. First, carbon credit incentives awarded for verifiable CO_2_ reductions can provide direct financial benefits. Second, income from the sale of value-added products enhances overall profitability and makes the integration of CCUS more attractive to industrial actors. This strategic combination increases the likelihood of voluntary adoption and strengthens the overall business case for CCUS within a regulatory compliance framework (Figure 5).

In contrast to global CCUS initiatives, India’s approach is uniquely adapted to its industrial and emissions landscape. More than 80% of the country’s CO_2_ emissions come from hard-to-abate sectors, making point-source capture from flue gases particularly relevant. This is in stark contrast to countries like the United States, where emissions are dispersed across various sectors and regions, making CDR technologies such as direct air capture more applicable. India’s targeted approach toward capturing CO_2_ at the source aligns better with the nature of its emissions and the economic structure of its industries. A strong example of India’s pragmatic approach is the operational carbon capture facility at Tuticorin Alkali Chemicals and Fertilizers Ltd. (TACFL) in Tamil Nadu, which captures approximately 60,000 tonnes of CO_2_ annually from flue gas using amine-based solvents, subsequently repurposing it for use in chemical manufacturing. While smaller in scale compared to international projects like Norway’s Sleipner or Canada’s Boundary Dam, which capture more than 1 million tonnes per annum (Mtpa) and are supported by extensive pipeline and geological storage infrastructure, India’s approach prioritizes cost-effective, modular solutions using abundant industrial byproducts like steel slag and fly ash. These materials offer considerable sequestration potential due to their high calcium and magnesium content, which can react with CO_2_ to generate stable carbonates. However, they require pre-treatment processes and are associated with slower reaction kinetics compared to conventional chemical capture methods. Still, India’s capture costs are highly competitive, often ranging from $40 to $50 per tonne of CO_2_, significantly lower than the $100 per tonne capture cost observed in solvent-based systems abroad. India possesses considerable geological storage potential within formations including basaltic rock, coal seams, and exhausted oil reservoirs. Nevertheless, the prevailing insufficiency of requisite infrastructure, such as CO_2_ transportation pipelines and extensive injection facilities, constitutes a significant obstacle to the comprehensive realization of carbon capture and storage initiatives. Despite this, the focus on circular material use, economic feasibility, and point-source capture offers a viable and scalable model that aligns with India’s development priorities and emission profile. Hence, the CCUS strategy of India is specifically designed to address its unique industrial and ecological challenges. By integrating both compulsory and voluntary policy mechanisms and emphasizing economically viable CO_2_ utilization pathways, India can position CCUS not only as a compliance tool but as a key component of sustainable industrial growth. This divergence approach from numerous international frameworks yet aligns seamlessly with India’s unique national context, facilitating the establishment of a climate-resilient future characterized by innovation, efficiency, and corporate responsibility.

### CCUS as a catalyst for economic growth of India

The deployment of CCUS technologies in India can serve as a catalyst for economic growth, particularly in hard-to-abate sectors. The steel, cement, chemical, and petrochemical sectors constitute a fundamental component of India’s industrial framework, and the decarbonization of these industries is essential for the realization of the nation’s net-zero objectives. CCUS offers a scalable solution to reduce GHG emissions while maintaining industrial productivity and competitiveness.

Moreover, by supporting the transition to blue hydrogen, CCUS can accelerate the development of a hydrogen economy in India. This transition aligns with global trends toward cleaner energy systems and positions India as a leader in low-carbon industrial innovation.

India’s ambitious climate goals under the Panchamrit^30^ framework necessitate a strategic approach to decarbonization, particularly in sectors heavily reliant on fossil fuels. While renewable energy adoption is a critical component, the integration of CCUS technologies offers a complementary pathway to achieve sustainable energy transitions. A robust CCUS policy framework that combines compulsory and voluntary mechanisms can incentivize industries to adopt carbon capture technologies while ensuring compliance with regulatory standards. Furthermore, the development of high-value end products through CCUS enhances its economic viability, creating new revenue streams and driving industrial innovation. By leveraging CCUS for applications such as blue hydrogen production, India can address the challenges of transitioning to a low-carbon economy while supporting its industrial base. This methodology not only conforms to India’s decarbonization objectives for the year 2030 but also establishes a framework for realizing net-zero emissions by the year 2070, thereby exemplifying a dedication to international climate governance and sustainable progress.

To support the advancement and practical deployment of CDR technologies in India, future research must focus on context-specific innovation, scalability, and integration. Given India’s industrial emission profile, greater emphasis should be placed on point-source capture strategies using low-cost, abundant materials, such as steel slag, fly ash, and basalt. Research should prioritize improving the reaction kinetics and carbon uptake capacity of these materials through pre-treatment methods, nano-structuring, or hybrid material development. Additionally, techno-economic assessments and life cycle analyses are essential to compare CDR solutions under Indian conditions. Pilot-scale demonstrations involving mineralization, biochar application, and CO_2_-derived product synthesis should be expanded to evaluate commercial viability. Another critical direction involves the development of decentralized capture systems tailored to small and medium-scale industries. Furthermore, integration of CDR pathways with renewable energy systems and the development of supportive digital infrastructure (e.g., monitoring, reporting, and verification (MRV) platforms) will be key to establishing credibility and tracking outcomes. Multidisciplinary collaborations across academia, industry, and policy institutions must be encouraged to accelerate R&D and commercialization. These research directions can help create a robust roadmap for India’s CDR sector and contribute meaningfully to national and global climate mitigation goals.

## Summary and future perspectives

The surging demand for energy, propelled by demographic expansion and industrial advancements, necessitates the development of innovative methodologies to reconcile economic progression with ecological sustainability. India’s reliance on fossil fuels, in conjunction with its pledge to attain net-zero emissions by the year 2070 and to diminish carbon intensity by 45% by 2030, highlights the pressing need for groundbreaking carbon management initiatives. CDR and CCU technologies offer viable pathways to address historical and ongoing CO_2_ emissions, particularly in hard-to-abate sectors such as power, steel, cement, and petrochemicals.

CDR technologies, with their potential to achieve carbon negativity, and CCU technologies, which enable carbon neutrality, must be implemented synergistically. India’s economic and demographic profile presents unique opportunities for CCU end products, particularly in the construction and agricultural sectors. CO_2_ mineralization, a permanent and stable storage method, exemplifies the potential for scalable and economically viable carbon utilization. Similarly, integrating captured CO_2_ in fertilizer production can revolutionize sustainable agriculture while addressing the environmental challenges of traditional and organic fertilizers.

Achieving the ambitious Panchamrit goals requires India to adopt robust CCUS policies that combine compliance mechanisms with voluntary incentives. Such policies can support industrial adoption of CCU technologies, enhance the market for CO_2_-derived products, and incentivize innovation. Deploying hybrid technologies that integrate both CDR and CCU solutions can further enhance efficiency and scalability, especially for the dual challenges of removing ambient and point-source CO_2_ emissions.

Future research on CDR in India should focus on solutions that are scalable, economically viable, and aligned with national development goals. Technologies such as mineralization, aggregate formation, and CO_2_ fixation in concrete hold immense promise not only for climate mitigation but also for supporting India’s growing infrastructure needs. In a country where large-scale construction and land development are priorities, mineralized CO_2_ can be used in building materials and landfilling applications, helping reclaim land while permanently storing carbon. Research must aim to optimize the reactivity and stability of mineralizing agents, improve aggregate binding properties, and ensure the durability of CO_2_-infused concrete. Moreover, integrating MRV (monitoring, reporting, and verification) components is essential to establish credibility and transparency. Future systems should include digital tracking of captured and utilized CO_2_, life cycle assessments of end products, and robust reporting frameworks. This will support the development of national CDR registries and facilitate participation in voluntary or compliance carbon markets. Pilot-scale demonstrations that combine technical validation with MRV implementation will be crucial to scale these technologies. By embedding MRV and focusing on material integration into mainstream construction, India can advance CDR strategies that are both environmentally effective and economically transformative.

In summary, India’s path to a sustainable future lies in the strategic implementation of CO_2_ mitigation technologies. These solutions not only align with India’s climate goals but also enable economic growth, industrial innovation, and energy security. By promoting public-private collaboration and leveraging its unique market potential, India can set a global precedent for achieving net-zero emissions while ensuring socioeconomic progress.

### Limitations of the study

The limitations of the study have been mentioned in the discussion section.

## Acknowledgments

The authors would like to acknowledge the support from DST, India-supported National Centre of Excellence in Carbon Capture and Utilization (DST/TMD/CCUS/10.13039/100031275CoE/202/IITB) for this research activity. The authors would also like to thank the Indian Institute of Technology Bombay (IITB) for the research support.

## Author contributions

P.M. and A.D. conceptualized the manuscript, and P.M. did the literature search and data analysis. P.M. and A.D. wrote the manuscript.

## Declaration of interests

The authors declare no conflict of interest.
